# Clinical features, diagnostic test performance, treatment and outcome of pulmonary tuberculosis patients with chronic pulmonary aspergillosis in China: a retrospective, observational study

**DOI:** 10.3389/fcimb.2025.1653842

**Published:** 2026-01-20

**Authors:** Jun Li, Naming Wu, Chunlin Mei, Minhui Mei, Shufang Chen, Chengqing Yang

**Affiliations:** 1 Wuhan Pulmonary Hospital, Wuhan Institute for Tuberculosis Control, Wuhan, Hubei, China; 2 Department of Dermatology, Renmin Hospital of Wuhan University, Wuhan, Hubei, China; 3 Department of Respiratory and Critical Care Medicine, Wuhan Pulmonary Hospital, Wuhan, Hubei, China

**Keywords:** chronic pulmonary aspergillosis, diagnosis, pulmonary tuberculosis, subtypes, treatment

## Abstract

**Introduction:**

Chronic pulmonary aspergillosis (CPA) is a major sequela of pulmonary tuberculosis (PTB), posing a significant health burden in high-prevalence regions like China. However, data on the clinical spectrum, diagnostic challenges, and outcomes of PTB-associated CPA in the Chinese population remain limited.

**Methods:**

This retrospective single-center study analyzed 220 patients with PTB-associated CPA in Wuhan Pulmonary Hospital (January-December 2022). CPA was diagnosed and subtyped according to European guidelines: simple aspergilloma (SA, n=31), chronic cavitary pulmonary aspergillosis (CCPA, n=120), chronic fibrosing pulmonary aspergillosis (CFPA, n=39), Aspergillus nodule (AN, n=25), and semi-invasive pulmonary aspergillosis (SAIA, n=5). Data pertaining to demographic and clinical characteristics, comorbidities, imaging findings, diagnostic test performance, antifungal treatment regimens, and clinical outcomes were retrospectively analyzed.

**Results:**

The cohort had a median age of 56.7 years, with a 64.1% male predominance. Cough (94.1%) and sputum (83.2%) were the most common symptoms, while hemoptysis was highest in CFPA (79.5%). Chest CT revealed cavities in 87.7% and a high prevalence of fibrosis in CFPA (89.7%). Serologically, serum Aspergillus IgG was positive in 68.2% of patients, with the highest positivity in CFPA (74.4%). BALF galactomannan positivity was highest in AN (76.0%). Voriconazole was the primary antifungal agent (69.1%), but 70.5% of patients received therapy for ≤6 months. Outcomes varied by subtype. CFPA had the worst prognosis (38.5% disease progression, 10.3% mortality), whereas AN patients demonstrated the highest clinical stability (92.0% stable disease).

**Conclusion:**

PTB-associated CPA in China exhibits distinct subtype characteristics. Accurate diagnosis requires a combination of modalities. Treatment responses vary by subtype, underscoring the need for region-specific clinical guidelines, multidisciplinary management, and further research on treatment duration, multi-center cohort studies, and improved diagnostic approaches.

## Introduction

Chronic pulmonary aspergillosis (CPA), a complex fungal infection caused by *Aspergillus* species (Denning et al., 2003), is strongly linked to preexisting lung conditions such as pulmonary tuberculosis (PTB), particularly in regions with high PTB prevalence (Baluku et al., 2021). A global analysis published in 2011 estimated that 852,000 to 1,372,000 individuals develop CPA within five years following a PTB diagnosis, with an annual rate of mortality or surgical resection ranging from 10% to 20% (Denning et al., 2011). Recent modeling by Denning et al. projected a global incidence of 1.8 million CPA cases annually, including 1.5 million prevalent cases among PTB-affected individuals and approximately 304,000 deaths per year (assuming 20% mortality in the first year and 7.5% in subsequent years) (Ikuta et al., 2024). Post-tuberculosis CPA is associated with a 7% one-year mortality rate, underscoring its clinical severity (Sengupta et al., 2025). China ranks third globally in PTB burden (Harris et al., 2019; Chen et al., 2024), and faces a substantial but insufficiently studied challenge from CPA. Structural lung damage caused by PTB, such as cavitation and fibrosis, provides a microenvironment that facilitates *Aspergillus* colonization (Ravimohan et al., 2018; Jaggi et al., 2024). Among PTB-related sequelae, CPA remains one of the most insidious and severe (Nam et al., 2010; Bongomin, 2020). Current evidence on CPA epidemiology, diagnostic accuracy, and treatment outcomes in Chinese PTB patients remains limited, impeding the development of targeted clinical management strategies.

Chronic cavitary pulmonary aspergillosis (CCPA) is the most common form of CPA and may progress to chronic fibrosing pulmonary aspergillosis (CFPA) if left untreated. Less common CPA subtypes include *Aspergillus* nodule (AN) and simple aspergilloma (SA), typically occurring in non-immunocompromised individuals with a history of current or prior pulmonary disease (Bongomin et al., 2020; Denning et al., 2016; Schweer et al., 2014). Semi-invasive aspergillosis (SAIA), previously known as chronic necrotizing pulmonary aspergillosis, represents a more rapidly progressive form of infection (typically <3 months in duration) and is more frequently observed in moderately immunocompromised patients (Denning et al., 2016; Chan et al., 2016). However, distinguishing CPA from active PTB or other fungal infections in post-PTB patients remains challenging due to overlapping clinical and radiological features. Delayed or inaccurate diagnosis may lead to progressive pulmonary damage, reduced treatment efficacy, and increased mortality, particularly in immunocompromised populations.

Long-term oral antifungal therapy can improve general health status and respiratory symptoms in patients with CPA, while controlling hemoptysis and preventing disease progression (Yagi et al., 2024; Cadena et al., 2021). However, significant challenges persist in clinical management. No consensus has been reached regarding optimal treatment regimens, and standardized definitions for treatment outcomes and endpoints are lacking. Prolonged antifungal administration is often required in most CPA patients. However, treatment efficacy is frequently limited by drug-induced adverse effects and the emergence of resistance, with approximately one-third of patients experiencing relapse after discontinuation (Koyama et al., 2014; Lamoth and Calandra, 2022). Current therapeutic strategies for CPA primarily rely on long-term administration of azole antifungals, such as voriconazole and itraconazole. Surgical intervention is generally reserved for patients with localized disease or life-threatening complications, including massive hemoptysis (Jaggi et al., 2024; Armstrong-James et al., 2023). Notably, in advanced phenotypes like CFPA, characterized by extensive fibrosis and poor responsiveness to medical therapy, treatment outcomes remain suboptimal (Kosmidis et al., 2017). Moreover, in resource-limited settings, additional challenges such as drug toxicity, interactions with antitubercular agents, and limited availability of novel antifungal drugs like isavuconazole further exacerbate the complexities of CPA management, underscoring the urgent need for innovative research and improved therapeutic approaches.

Clinical studies investigating CPA in the context of PTB in China remain limited, and the current understanding of CPA in this population is insufficient. This retrospective single-center study aims to characterize the clinical features, diagnostic test performance, treatment approaches, and outcomes of PTB patients with CPA, including those who have completed therapy. By addressing these knowledge gaps, the study seeks to generate evidence-based insights to guide the optimization of CPA management in high-PTB-burden settings, ultimately contributing to improved patient outcomes and the development of targeted public health policies.

## Materials and methods

### Study design

This retrospective study was conducted from January 2022 to December 2022 at Wuhan Pulmonary Hospital. The study included patients diagnosed with inactive PTB (post-tuberculosis lung disease) complicated by CPA. All PTB cases were confirmed to have achieved clinical cure through standard anti-tuberculosis treatment, with a diagnosis of inactive PTB made at least 2 years prior to the CPA diagnosis; no evidence of active PTB lesions was observed on chest imaging, and sputum smear/culture results were negative at the time of CPA diagnosis. Patients with active PTB or concurrent active PTB and CPA were excluded. Among the included patients, 100% had a history of PTB (post-tuberculosis lung disease), and 0% had active PTB at the time of CPA diagnosis. All CPA diagnoses were independently reviewed and confirmed by at least two senior respiratory medicine specialists.

### Study population

PTB was diagnosed using sputum smear microscopy, culture, or GeneXpert detection of Mycobacterium tuberculosis from clinical specimens. CPA diagnosis and classification were based on criteria outlined in the European guidelines (Denning et al., 2016), and included the following: (1) Chest computed tomography (CT) findings consistent with CPA imaging features. (2) Microbiological evidence or immunological evidence of Aspergillus infection, defined by at least one of the following: (a) positive fungal culture for Aspergillus on sputum or bronchoscopic lavage fluid (BALF); (b) positive nucleic acid for Aspergillus on sputum or BALF; (c) positive galactomannan (GM) antigen test on BALF; (d) positive serum Aspergillus IgG; (e) Aspergillus infection on histopathology of the lung. (3) Clinical, radiological, and microbiological features persisting for at least three months.

Exclusion criteria included: (1) Absence of a prior history of PTB (excluding patients with no PTB history). (2) Active PTB or concurrent active PTB and CPA. (3) Diagnosis of invasive pulmonary aspergillosis. (4) Diagnosis of allergic bronchopulmonary aspergillosis. (5) Presence of non-Aspergillus fungal infections. (6) Clinically suspected but unconfirmed Aspergillus infection.

### Evaluation of clinical response

1. Stable: 1) Following surgery and/or antifungal therapy for ≥3 months, respiratory symptoms were significantly relieved, respiratory fungal cultures were negative, and chest imaging showed improvement. Specifically, non-cavitary lesions were reduced by ≥50% (persistently stable dense nodules ≤1 cm were excluded), cavity wall or adjacent pleural thickness (baseline ≥2 mm) was reduced by ≥20%, and fungal balls or bands had resolved. No imaging deterioration was observed in other regions, and no new lesions suspicious for CPA appeared within 6 months postoperatively. 2) Following surgery and/or antifungal therapy for ≥3 months, respiratory symptoms improved, and chest imaging remained stable without progression. 3) No antifungal treatment was performed, and the symptoms and imaging were stable within 6 months, and the microbiological evidence of respiratory tract Aspergillosis was negative.

2. Worsened: 1) In patients receiving surgery and/or antifungal therapy, clinical symptoms worsened, chest imaging showed deterioration or respiratory specimens remained positive for Aspergillus at 3 and 6 months, with other causes of deterioration excluded. 2) clinical symptoms or chest imaging findings worsened by 6 months in patients not treated with antifungals.

3. Death: Mortality was defined as all-cause death occurring within one year of follow-up.

### Laboratory procedures

Sputum samples were inoculated onto Sabouraud dextrose agar and BALF samples onto Sabouraud glucose agar, followed by incubation at 25°C and 35°C for 2–7 days. Fungal colonies were examined daily. The presence of characteristic *Aspergillus s*tructures, such as conidiophores and foot cells, indicated *Aspergillus* growth. Species identification was primarily based on macroscopic colony morphology and microscopic characteristics. Nucleic acid detection of *Aspergillus* was performed using real-time PCR (Dilan, China), with a cycle threshold (Ct) value ≤36 considered positive. GM antigen testing in serum or BALF was performed using enzyme-linked immunosorbent assay (ELISA; Bio-Rad Laboratories, CA, USA) according to the manufacturer’s instructions. For diagnostic interpretation, a GM index (GMI) ≥0.5 was defined as positive for serum, while a more specific threshold of GMI ≥0.8 was adopted for BALF, in accordance with current guidelines (Salzer et al., 2018; de Oliveira et al., 2023). *Aspergillus*-specific IgG antibody testing was conducted using ELISA (Dynamiker, China). It is important to note that this assay uses *Aspergillus* galactomannan as the plate-coating antigen. Based on the manufacturer’s instructions and for the purpose of this study, a value ≥80 AU/mL was considered positive (with the equivocal range of 80–120 AU/mL included in the positive group for analysis).

### Statistical analysis

Quantitative variables were expressed as mean ± standard deviation (SD) for normally distributed data or as median with interquartile range (IQR) for non-normally distributed data. Categorical variables were presented as counts and percentages. Comparisons between groups were performed using the Student’s t-test for normally distributed variables and the Mann–Whitney U test for non-normally distributed variables. Significance was set at *P* <0.05. GraphPadPrism 9 (GraphPad Software, La Jolla, CA, USA) was utilized to analyze data.

## Results

### General characteristics

A total of 220 PTB patients with CPA were included in the study. The cohort comprised 31 with SA, 120 with CCPA, 39 with CFPA, 25 with AN, and 5 with SAIA. Patient general characteristics are summarized in **Table 1**. The study cohort had a mean age of 56.7 ± 14.7 years (range: 21–84 years), with a significant male predominance (141 males, 79 females). Male patients were the majority in the CFPA, CCPA, and SA subgroups. Patients with CFPA and CCPA were older compared to those in other subtypes.

**Table 1:** Baseline characteristics of pulmonary tuberculosis (PTB) patients with chronic pulmonary aspergillosis (CPA) in different subtypes

Characteristic	ALL (*n* = 220)	SA (*n* = 31)	CCPA (*n* = 120)	CFPA (*n* = 39)	AN (*n* = 25)	SAIA (*n* = 5)
Sex, male	141 (64.1)	20 (64.5)	81 (67.5)	29 (74.4)	9 (36.0)	2 (40.0)
Age, years	56.7 ± 14.7	54.6 ± 14.8	58.5 ± 14.0	59.1 ± 12.2	47.2 ± 17.7	53.4 ± 15.4
Underlying disease, *n* (%)						
Bronchial TB	56 (25.5)	2 (6.5)	29 (24.2)	14 (35.9)	9 (36.0)	2 (40.0)
Lung tumor	2 (0.9)	0 (0.0)	1 (0.8)	1 (2.6)	0 (0.0)	0 (0.0)
Bronchiectasis	31 (14.1)	0 (0.0)	22 (18.3)	5 (12.8)	3 (12.0)	1 (20.0)
Pneumoconiosis	9 (4.1)	1 (3.2)	7 (5.8)	0 (0.0)	1 (4.0)	0 (0.0)
COPD	49 (22.3)	6 (19.4)	32 (26.7)	9 (23.1)	1 (4.0)	1 (20.0)
Pulmonary sarcoidosis	0 (0.0)	0 (0.0)	0 (0.0)	0 (0.0)	0 (0.0)	0 (0.0)
Interstitial lung disease	4 (1.8)	2 (6.5)	2 (1.7)	0 (0.0)	0 (0.0)	0 (0.0)
Diabetes	44 (20.0)	8 (25.8)	26 (21.7)	7 (17.9)	3 (12.0)	0 (0.0)
Use of glucocorticoid	10 (4.5)	2 (6.5)	7 (5.8)	0 (0.0)	1 (4.0)	0 (0.0)

PTB, pulmonary tuberculosis; CPA, chronic pulmonary aspergillosis; SA, simple aspergilloma; CCPA, chronic cavitary pulmonary aspergillosis; CFPA, chronic fibrosing pulmonary aspergillosis; AN, Aspergillus nodule; SAIA, semi-invasive aspergillosis; COPD, chronic obstructive pulmonary disease.

Most patients presented with pre-existing pulmonary or systemic comorbidities in addition to PTB. The most prevalent conditions were bronchial tuberculosis (*n* = 56, 25.5%), chronic obstructive pulmonary disease (COPD, *n* = 49, 22.3%), and diabetes mellitus (*n* = 44, 20.0%), followed by bronchiectasis (*n* = 31, 14.4%), interstitial lung disease (*n* = 4, 1.8%), and lung tumor (0.9%, *n* = 2). A diagnosis of bronchial tuberculosis was based on either a documented prior history (self-reported or bronchoscopy-confirmed records) or characteristic chest CT findings such as bronchial wall thickening and luminal stenosis. Among subgroup analyses, bronchial tuberculosis was the most common comorbidity in the AN (36.0%, *n* = 9) and SAIA (*n* = 2, 40.0%) subgroups, while COPD was frequently observed in CCPA (*n* = 32, 26.7%) and CFPA (*n* = 9, 23.1%). Diabetes mellitus was more prevalent in SA (*n* = 8, 25.8%) and CCPA (*n* = 26, 21.7%).

Ten patients had a history of prolonged glucocorticoid use, including seven with CCPA, two with SA, and one with AN. Two cases of lung cancer were identified, affecting one patient each in the CCPA and CFPA subgroups. Interstitial lung disease and pneumoconiosis were primarily observed in the SA and CCPA subgroups, while no cases of pulmonary sarcoidosis were detected.

### Symptoms and radiological examinations

Clinical and radiological data are summarized in **Table 2**. The most frequently reported symptoms were cough (*n* = 207, 94.1%) and sputum production (*n* = 183, 83.2%), followed by hemoptysis (*n* = 126, 57.3%) and fatigue (*n* = 45, 20.5%). These symptoms were most prevalent in the CCPA, CFPA, and SAIA subgroups. Hemoptysis occurred more frequently in CFPA (79.5%) and SA (71.5%) than in CCPA (55.8%). Fever was predominantly observed in AN (55.8%) and SAIA (40.0%). Notably, fever was observed in 2 of the 5 (40.0%) SAIA patients; however, given the very small sample size (n = 5) of this subgroup, this finding must be interpreted with caution and cannot reliably support comparative claims regarding fever rates across subtypes. CFPA patients exhibited significantly higher incidences of dyspnea (51.3%), chest pain (28.2%), and weight loss (53.8%) compared to other groups.

**Table 2:** The clinical and radiological manifestation of PTB patients with CPA in different subtypes.

Clinical feature	ALL (*n* = 220)	SA (*n* = 31)	CCPA (*n* = 120)	CFPA (*n* = 39)	AN (*n* = 25)	SAIA (*n* = 5)
Clinical manifestations, *n* (%)						
Cough	207 (94.1)	24 (77.4)	118 (98.3)	39 (100.0)	21 (84.0)	5 (100.0)
Sputum	183 (83.2)	18 (58.1)	112 (93.3)	38 (97.4)	10 (40.0)	5 (100.0)
Hemoptysis	126 (57.3)	22 (71.0)	67 (55.8)	31 (79.5)	5 (20.0)	1 (20.0)
Fatigue	45 (20.5)	5 (16.1)	16 (13.3)	18 (46.2)	3 (12.0)	3 (60.0)
Fever	22 (10.0)	2 (6.5)	12 (10.0)	5 (12.8)	1 (4.0)	2 (40.0)
Dyspnea	77 (35.0)	5 (16.1)	44 (36.7)	20 (51.3)	6 (24.0)	2 (40.0)
Chest pain	26 (11.8)	4 (12.9)	7 (5.8)	11 (28.2)	4 (16.0)	0 (0.0)
Weight loss	60 (27.3)	6 (19.4)	27 (22.5)	21 (53.8)	4 (16.0)	2 (40.0)
Imaging manifestations, *n* (%)						
Right lung	162 (73.6)	19 (61.3)	100 (83.3)	18 (46.2)	20 (80.0)	5 (100.0)
Left lung	132 (60.0)	13 (41.9)	67 (55.8)	35 (89.7)	14 (56.0)	3 (60.0)
Lung field	218 (99.1)	31 (100.0)	119 (99.2)	39 (100.0)	24 (96.0)	5 (100.0)
Cavity	193 (87.7)	31 (100.0)	120 (100.0)	39 (100.0)	1 (4.0)	2 (40.0)
Air crescent sign	147 (66.8)	31 (100.0)	77 (64.2)	38 (97.4)	0 (0.0)	1 (20.0)
Intracavity septum	166 (75.5)	8 (25.8)	116 (96.7)	39 (100.0)	0 (0.0)	3 (60.0)
Progressive pleural thickening	166 (75.5)	16 (51.6)	108 (90.0)	39 (100.0)	1 (4.0)	2 (40.0)
Emphysema	121 (55.0)	8 (25.8)	71 (59.2)	31 (79.5)	9 (36.0)	2 (40.0)
Fibrosis	129 (58.6)	8 (25.8)	76 (63.3)	35 (89.7)	9 (36.0)	1 (20.0)
Upper lung	189 (85.9)	27 (87.1)	106 (88.3)	37 (94.9)	15 (60.0)	4 (80.0)
Lower lung	45 (20.5)	3 (9.7)	18 (15.0)	10 (25.6)	12 (48.0)	2 (40.0)

PTB, pulmonary tuberculosis; CPA, chronic pulmonary aspergillosis; SA, simple aspergilloma; CCPA, chronic cavitary pulmonary aspergillosis; CFPA, chronic fibrosing pulmonary aspergillosis; AN, Aspergillus nodule; SAIA, semi-invasive aspergillosis.

Chest CT imaging was abnormal in all enrolled patients. The lesions primarily involved the upper lung zones (85.9%), with distribution across the right lung (162 patients), left lung (132 patients), and lung field (218 patients), consistent with bilateral disease in a significant portion of the cohort. Dominant CT abnormalities included cavity formation (87.7%), intracavitary septum (75.5%), progressive pleural thickening (75.5%), fibrosis (58.6%), and emphysema (55.0%). Cavity formation was universal in SA, CCPA, and CFPA but rare in AN (*n* = 1) and SAIA (*n* = 2). CFPA showed significantly higher rates of emphysema (79.5%) and fibrosis (89.7%) than other subtypes, reflecting advanced fibrotic remodeling.

**Figure 1:**
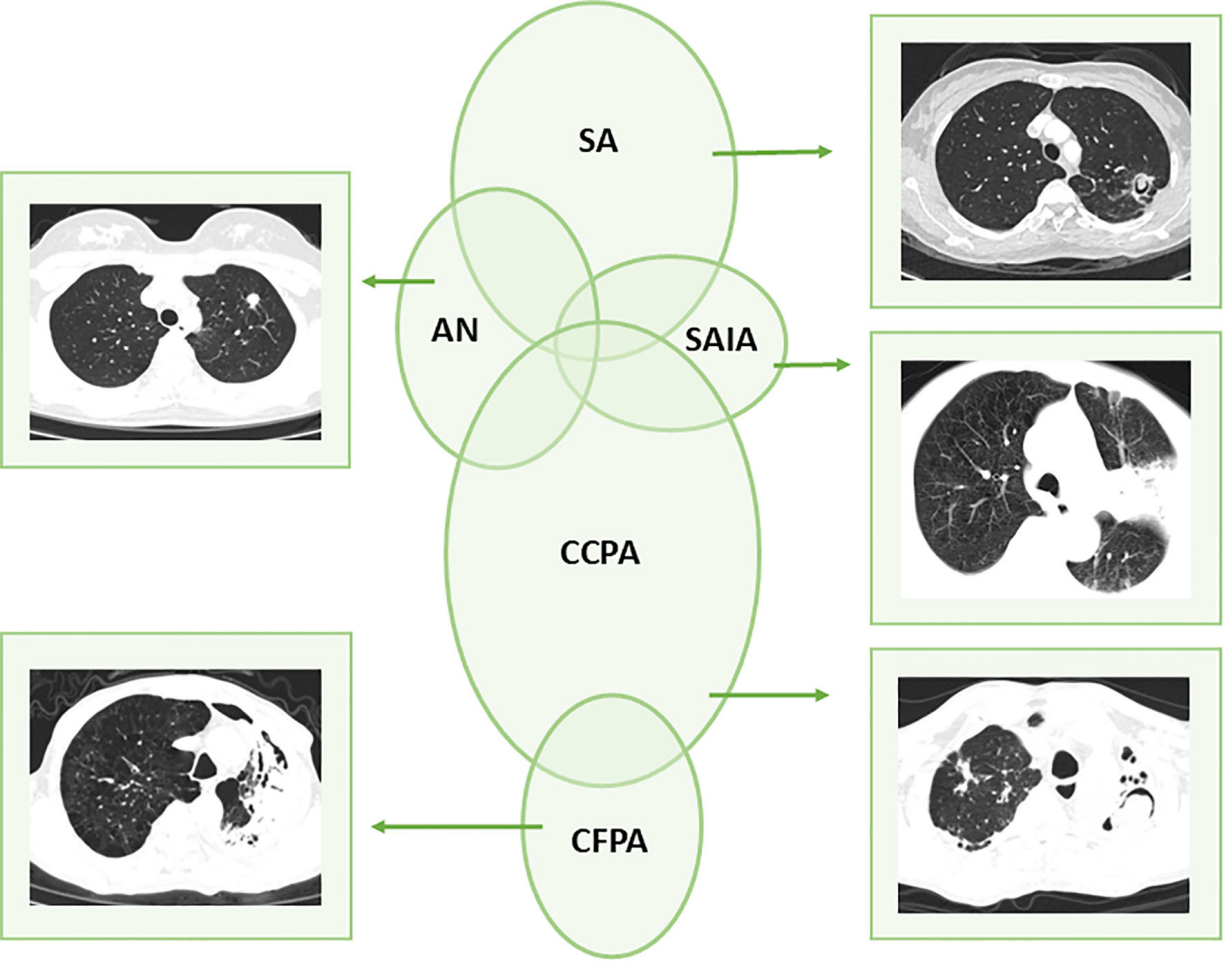
Chronic pulmonary aspergillosis (CPA) classification on high-resolution computed tomography at diagnosis. SA, simple aspergilloma; CCPA, chronic cavitary pulmonary aspergillosis; CFPA, chronic fibrosing pulmonary aspergillosis; AN, *Aspergillus* nodule; SAIA, semi-invasive aspergillosis.

High-resolution computed tomography (HRCT) findings at the time of diagnosis are presented in **Figure 1**. All CT/HRCT images were independently reviewed by two board-certified radiologists with ≥5 years of experience in thoracic imaging; discrepancies were resolved by consensus with a senior thoracic radiologist (≥10 years of experience) blinded to clinical and microbiological data. Radiologic assessments and subtype definitions strictly followed the ESCMID/ERS guidelines (Denning et al., 2016), with core imaging features defining each subtype: AN presents as one or more nodules (with or without cavitation), frequently showing necrosis and mimicking radiological features of tuberculoma or lung carcinoma; CCPA, the most prevalent subtype, is characterized by one or more thin- or thick-walled pulmonary cavities, potentially containing aspergillomas or irregular intraluminal material, along with overt radiological progression (new cavities, increasing pericavitary infiltrates, or progressive fibrosis) over at least 3 months; CFPA manifests as severe fibrotic destruction involving at least two lung lobes (with focal fibrotic cavitation in a single lobe classified as CCPA), typically presenting as consolidation or large cavities surrounded by fibrosis; SA is defined by a solitary pulmonary cavity containing a fungal ball and no radiological progression over ≥3 months; and SAIA exhibits variable imaging features including cavitation, nodules, and progressive consolidation with abscess formation. CT imaging plays a critical role in the rapid evaluation of CPA, particularly in cases where microbiological confirmation is delayed. Radiological phenotypes varied across the CPA spectrum but consistently demonstrated upper lobe predominance. Overlapping radiological features were frequently observed (e.g., CCPA-AN/SAIA transitions), supporting the concept of CPA as a clinical-radiological continuum rather than a set of distinct entities consistent with its progressive pathological nature.

### Laboratory examinations

**Figure 2:**
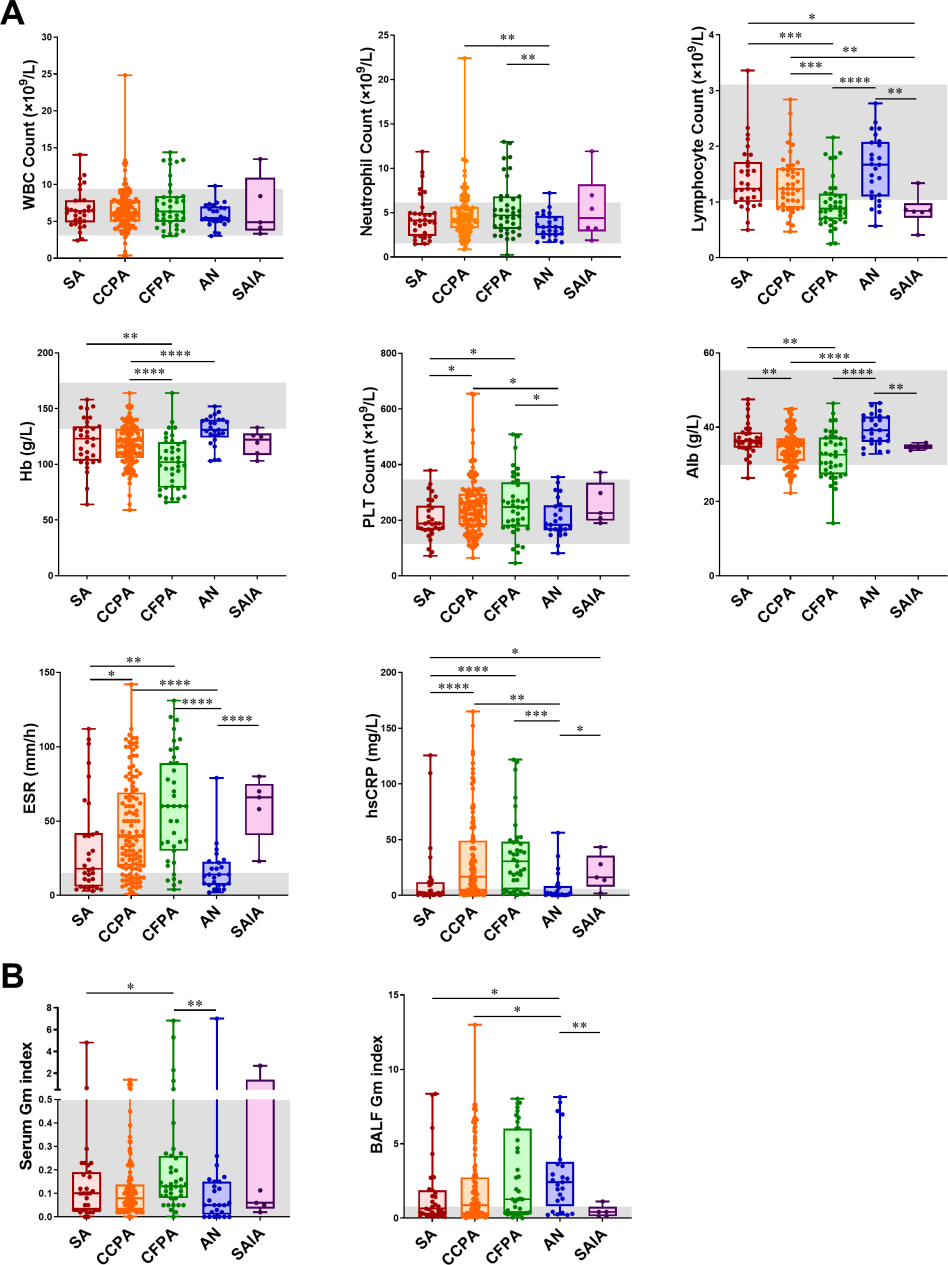
Laboratory test results of pulmonary tuberculosis (PTB) patients with CPA in different subtypes. **(A)** Blood routine results of patients with different CPA subtypes. **(B)** Galactomannan results in serum and bronchoalveolar lavage fluid (BALF) of patients with different CPA subtypes. WBC, white blood cell; Hb, hemoglobin; PLT, blood platelet; Alb, albumin; ESR, erythrocyte sedimentation rate; CRP, C-reactive protein; GM, galactomannan; BALF, bronchoalveolar lavage fluid. The gray area represents the normal range. * p < 0.05, ** p < 0.01, *** p < 0.001 and **** p < 0.0001.

Laboratory and microbiological data are presented in **Table 3**, with subtype-specific variations illustrated in **Figure 2**. All 220 CPA patients had direct evidence of *Aspergillus* infection. Most patients exhibited normal white blood cell (WBC) counts (median 6.2×10^9^/L, IQR 4.9-7.8) and neutrophil counts (median 4.2×10^9^/L, IQR 3.1-5.6), though CFPA (median 4.7×10^9^/L) and CCPA (4.3×10^9^/L) showed significantly higher neutrophilia than AN (3.4×10^9^/L, *P* < 0.01 for both). Lymphocyte counts were below normal in SAIA (0.8×10^9^/L) and CFPA (0.9×10^9^/L), while AN had higher counts (1.7×10^9^/L) compared to CCPA (1.2×10^9^/L) and SA (1.2×10^9^/L), remaining within physiological limits. The low lymphocyte count in the SAIA subgroup (n = 5) is noted but requires validation in larger cohorts. Hypochromic anemia was common across the cohort, with a median hemoglobin (Hb) level of 118 g/L. Anemia was most severe in CFPA (102 g/L), while SA (123 g/L) and CCPA (117 g/L) had significantly lower Hb compared to AN (131 g/L; *P* < 0.05). Platelet (PLT) counts were generally within normal limits (median: 225 × 10^9^/L), though mild thrombocytosis was noted in CCPA (239 × 10^9^/L) and CFPA (247 × 10^9^/L). Hypoalbuminemia was more pronounced in CFPA (27 g/L) and CCPA (31 g/L) compared to other subtypes (*P* < 0.05). Inflammatory markers were elevated across the cohort. The erythrocyte sedimentation rate (ESR) showed a median value of 35 mm/h, with the highest levels observed in SAIA (66 mm/h), followed by CFPA (60 mm/h). High-sensitivity C-reactive protein (hsCRP) levels were also elevated, peaking in CFPA (30.6 mg/L) and followed by CCPA (16.8 mg/L).

**Table 3:** Laboratory and microbiology data of PTB patients with CPA in different subtypes.

Variable	ALL (*n* = 220)	SA (*n* = 31)	CCPA (*n* = 120)	CFPA (*n* = 39)	AN (*n* = 25)	SAIA (*n* = 5)
Laboratory data, median (IQR)						
WBC count, 10^9^/L	6.2 (4.9-7.8)	6.4 (4.9-7.9)	6.2 (5.0-7.9)	6.3 (4.9-8.4)	5.5 (5.0-7.0)	4.9 (3.8-11.0)
Neutrophil count, 10^9^/L	4.2 (3.01-5.57)	4.1 (2.4-5.0)	4.3 (3.3-5.7)	4.7 (3.2-6.8)	3.4 (2.5-4.6)	4.4 (2.9-8.2)
Lym count, 10^9^/L	1.2 (0.9-1.7)	1.2 (1.0-1.7)	1.2 (0.9-1.6)	0.9 (0.7-1.2)	1. 7 (1.1-2.1)	0.8 (0.7-1.0)
HB, g/L	118 (103-132)	123 (103-134)	117 (106-132)	102 (79-120)	131 (124-140)	122 (108-128)
PLT count, 10^9^/L	225 (173-288)	188 (166-252)	239 (182-295)	247 (117-336)	184 (164-254)	226 (199-335)
ALB, g/L	35.4 (31.6-38.3)	36.3 (34.5-38.6)	34.9 (31.0--37)	32.6 (27.3-37.2)	39.2 (36.0-42.9)	34.5 (34.1-35.4)
ESR, mm/h	35 (14-67)	18 (6-42)	40 (20-69)	60 (30-89)	14 (7-23)	66 (41-75)
hsCRP, mg/L	13.4 (1.9-40.0)	2.1 (0.8-11.9)	16.8 (3.9-49.1)	30.6 (5.3-48.3)	1.4 (0.4-8.3)	16.2 (7.9-35.6)
Microbiology data, *n* (%)						
Fungal smear	22 (10.0)	3 (9.7)	16 (13.3)	2 (5.1)	0 (0.0)	1 (20.0)
Fungal culture	54 (24.5)	7 (22.6)	28 (23.3)	14 (35.9)	4 (16.0)	1 (20.0)
Serum GM	16 (7.3)	2 (6.5)	7 (5.8)	5 (12.8)	1 (4.0)	1 (20.0)
BALF GM	135 (61.4)	17 (54.8)	72 (60.0)	26 (66.7)	19 (76.0)	1 (20.0)
NGS or PCR	119 (54.1)	18 (58.1)	63 (52.5)	16 (41.0)	18 (72.0)	4 (80.0)
Aspiration biopsy	1 (0.5)	0 (0.0)	0 (0.0)	0 (0.0)	0 (0.0)	1 (20.0)
Bronchoscopy	6 (2.7)	1 (3.2)	0 (0.0)	1 (2.6)	2 (8.0)	2 (40.0)
Surgical excision	43 (19.5)	13 (41.9)	21 (17.5)	8 (20.5)	1 (4.0)	0 (0.0)
Aspergillus specfic IgG antibody, *n*	150 (68.2)	15 (48.4)	87 (72.5)	29 (74.4)	18 (72.0)	1 (20.0)

PTB, pulmonary tuberculosis; CPA, chronic pulmonary aspergillosis; SA, simple aspergilloma; CCPA, chronic cavitary pulmonary aspergillosis; CFPA, chronic fibrosing pulmonary aspergillosis; AN, Aspergillus nodule; SAIA, semi-invasive aspergillosis; IQR, interquartile range; WBC, white blood cell; Lym, lymphocyte; PLT, blood platelet; HB, hemoglobin; ALB, albumin; ESR, erythrocyte sedimentation rate; CRP, C-reactive protein; GM, galactomannan; BALF, bronchoalveolar lavage fluid; NGS, next generation sequencing; PCR, polymerase chain reaction.

Pathological confirmation was obtained using next-generation sequencing (NGS) or polymerase chain reaction (PCR) in 119 patients. Additional diagnostic methods included aspiration biopsy (*n* = 1), bronchoscopy (*n* = 6), and surgical excision (*n* = 43). The overall fungal smear positivity rate was 10.0% (*n* = 22). Culture positivity varied by subtype: CFPA (35.9%), CCPA (23.3%), SA (22.6%), SAIA (20.0%), and AN (16.0%). Serum GM index levels were predominantly within normal ranges, whereas BALF GM index more frequently exceeded diagnostic thresholds. BALF GM positivity was highest in AN (76.0%), significantly exceeding SA (41.9%), CCPA (52.5%), and SAIA (20.0%, *P* < 0.05). Serum *Aspergillus*-specific IgG was positive in 68.2% of patients, with CFPA (74.4%), CCPA (72.5%), and AN (72.0%) showing higher rates than SA (48.4%) and SAIA (20.0%). The low rates of BALF GM positivity and serum IgG positivity in the SAIA subgroup (n = 5) are presented; however, the very small sample size limits any conclusive interpretation of these findings.

**Figure 3:**
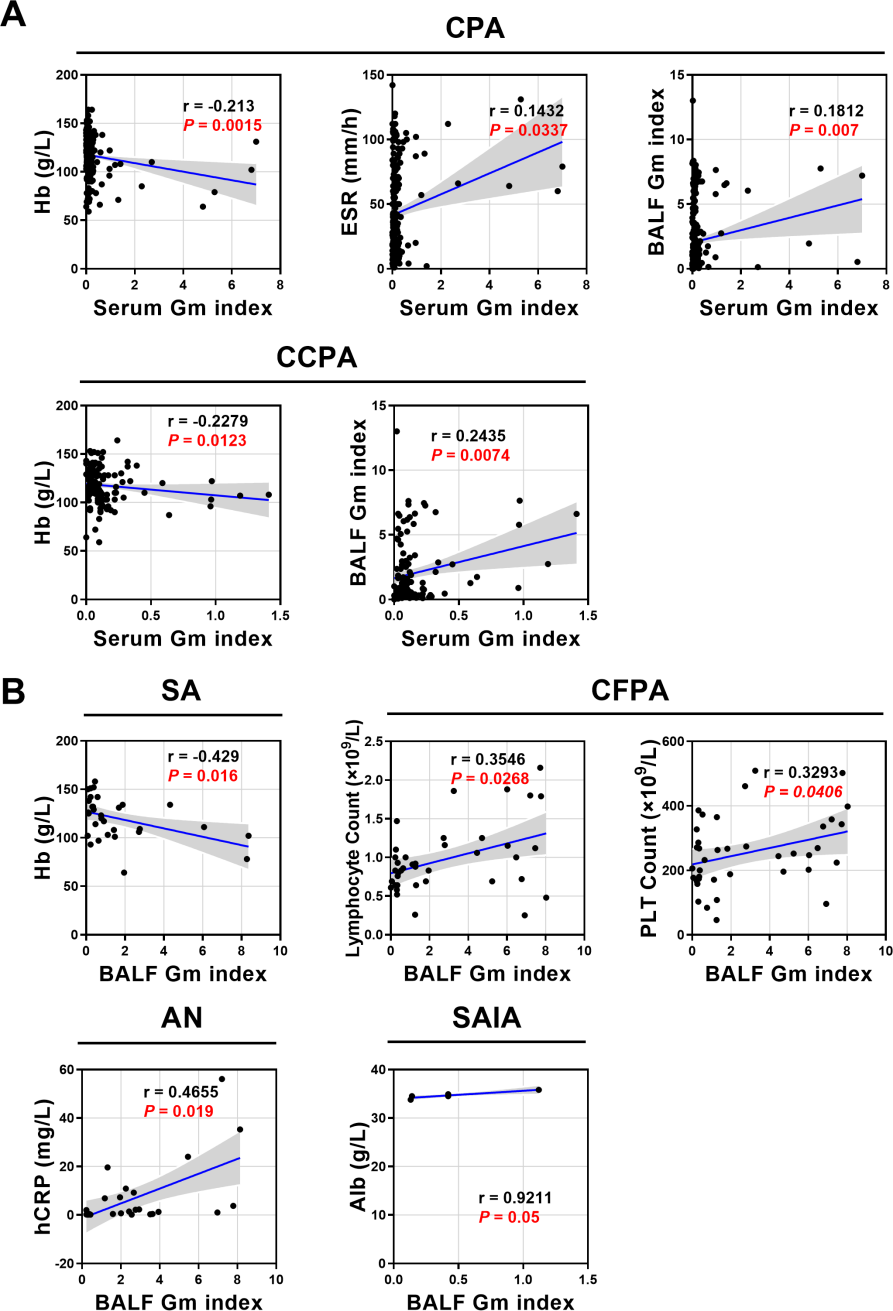
Correlation analysis of GM index with laboratory parameters across CPA subtypes. **(A)** Correlations between serum GM index and hematologic indicators in overall CPA and CCPA subtypes. **(B)** Correlations between BALF GM index and blood parameters in SA, CFPA, AN, and SAIA subtypes.

Given the critical role of GM in CPA, correlations between GM levels and laboratory parameters were further analyzed (**Figure 3**; **Supplementary Figures S1–12**). Overall, serum GM in CPA patients showed a negative correlation with Hb and a positive correlation with both ESR and BALF GM. No significant associations were found between BALF GM and peripheral blood parameters, possibly due to limited systemic GM release. Subtype-specific correlation patterns revealed further distinctions. In CCPA, serum GM negatively correlated with Hb and positively correlated with BALF GM. In SA, BALF GM negatively correlated with Hb. In AN, BALF GM was positively associated with hsCRP, with no other significant correlations. In CFPA, BALF GM positively correlated with lymphocytes and PLT. Finally, only a positive correlation between BALF GM and Alb was detected in SAIA. Notably, the SAIA subgroup included only 5 patients, so this correlation should be interpreted with caution due to the small sample size.

### Treatment and clinical outcomes

As outlined in **Table 4**, antifungal therapy was administered to 90.0% (198 of 220) CPA patients. Among them, 155 received pharmacologic treatment alone, while 43 underwent combined antifungal therapy and surgical intervention. Voriconazole was the most frequently prescribed agent (*n* = 152, 69.1%), followed by itraconazole (*n* = 13, 5.9%) and amphotericin B (*n* = 3, 1.4%). Adverse drug reactions (ADRs) were reported in 19 patients, with the highest incidence observed in the AN subgroup (*n* = 4, 16%). Most patients (*n* = 155, 70.5%) received antifungal therapy for ≤6 months, whereas 57 patients did not undergo any antifungal treatment—primarily due to economic constraints preventing the affordability of antifungal agents or follow-up observation recommended for asymptomatic patients with radiologically stable CT lesions following clinical evaluation. Only eight patients continued treatment beyond six months, including 1 SA, 5 CCPA, and 2 CFPA cases.

**Table 4:** Treatment and outcomes of PTB patients with CPA in different subtypes

Variable	ALL (*n* = 220)	SA (*n* = 31)	CCPA (*n* = 120)	CFPA (*n* = 39)	AN (*n* = 25)	SAIA (*n* = 5)
Treatment method, *n* (%)						
Drug alone	155 (70.5)	20 (64.5)	86 (71.7)	25 (64.1)	19 (76.0)	5 (100.0)
Surgery alone	0 (0.0)	0 (0.0)	0 (0.0)	0 (0.0)	0 (0.0)	0 (0.0)
Drug + surgery	43 (19.5)	13 (41.9)	21 (17.5)	8 (20.5)	1 (4.0)	0 (0.0)
Antifungal drug, *n* (%)						
Itraconazole	13 (5.9)	0 (0.0)	7 (5.8)	3 (7.7)	1 (4.0)	2 (40.0)
Voriconazole	152 (69.1)	20 (64.5)	81 (67.5)	30 (76.9)	18 (72.0)	3 (60.0)
Amphotericin B	3 (1.4)	0 (0.0)	2 (1.7)	1 (2.6)	0 (0.0)	0 (0.0)
Posaconazole	0 (0.0)	0 (0.0)	0 (0.0)	0 (0.0)	0 (0.0)	0 (0.0)
**Never antifungal treatment, *n* (%)**	57 (25.9)	11 (35.5)	34 (28.3)	6 (15.4)	6 (24.0)	0 (0.0)
**Adverse drug reactions, *n* (%)**	19 (8.6)	3 (9.7)	7 (5.8)	5 (12.8)	4 (16.0)	0 (0.0)
Antifungal time, month, *n* (%)						
≤6	155 (70.5)	19 (61.3)	81 (67.5)	31 (79.5)	19 (76.0)	5 (100.0)
>6	8 (3.6)	1 (3.2)	5 (4.2)	2 (5.1)	0 (0.0)	0 (0.0)
Clinical response, *n* (%)						
Stable	167 (75.9)	24 (77.4)	96 (80.0)	20 (51.3)	23 (92.0)	4 (80.0)
Worsened	49 (22.3)	7 (22.6)	24 (20.0)	15 (38.5)	2 (8.0)	1 (20.0)
Mortality	4 (1.8)	0 (0.0)	0 (0.0)	4 (10.3)	0 (0.0)	0 (0.0)

PTB, pulmonary tuberculosis; CPA, chronic pulmonary aspergillosis; SA, simple aspergilloma; CCPA, chronic cavitary pulmonary aspergillosis; CFPA, chronic fibrosing pulmonary aspergillosis; AN, Aspergillus nodule; SAIA, semi-invasive aspergillosis.

Clinical response evaluation revealed that 167 patients (75.9%) achieved stable disease, while 49 (22.3%) experienced disease progression. Four patients (10.3%) in the CFPA subgroup died during follow-up, with no deaths directly attributable to CPA, massive hemoptysis, or fungal sepsis. Adjudicated causes included surgery-related severe pneumonia, advanced malignancy with severe pneumonia, drug-induced multiple organ failure, and COPD acute exacerbation with respiratory failure—consistent with the subgroup’s baseline characteristics (older age, lower hemoglobin and albumin, higher rates of COPD and malignancy). CFPA patients had the poorest overall outcomes, with a significantly lower stable disease rate (51.3%) and the highest progression rate (38.5%) among all subtypes. In contrast, AN patients showed the most favorable outcomes, with the highest stable disease rate (92.0%) and the lowest progression rate (8.0%).

## Discussion

PTB-associated CPA represents a complex fungal infection with significant clinical implications. However, its epidemiological patterns, diagnostic performance, mechanisms of antifungal resistance, and optimal therapeutic approaches remain inadequately defined. In this retrospective analysis of 220 Chinese patients with PTB-associated CPA, five distinct CPA subtypes were systematically evaluated to characterize clinical features, assess diagnostic methods, and determine treatment outcomes.

The predominance of CCPA (54.5% of cases) aligns with its established role as the most common CPA subtype in high-PTB-burden regions, where PTB-induced lung cavitation provides a favorable microenvironment for *Aspergillus* colonization (Niu et al., 2020; Nam et al., 2023). The observed male predominance (64.1%) and the median age of 56.7 years are in line with global trends, potentially reflecting the higher burden of PTB and the chronic disease course that more commonly affects older individuals (Li et al., 2022; Jhun et al., 2013).

Notably, CFPA and CCPA were associated with older age and comorbidities such as COPD (22.3%) and bronchial tuberculosis (25.5%), whereas SAIA showed a strong link to bronchial TB (40%) and systemic inflammation (elevated hsCRP, and ESR). These patterns differ from those observed in low-PTB-burden regions like France (Maitre et al., 2021), where COPD/emphysema (44%) represent the predominant risk factors, highlighting the influence of regional PTB epidemiology on CPA pathogenesis. The serum GM index was lower than the BALF GM index, likely due to the localized release of GM antigens at the site of pulmonary infection with limited systemic dissemination. The AN subgroup was characterized by universal radiological evidence of aspergilloma and the highest BALF GM positivity rate (76%), consistent with its localized disease pattern in contrast to the more invasive nature of other CPA subtypes.

Diagnostically, the study highlights the subtype-specific performance of serological and microbiological tests. *Aspergillus*-specific IgG antibody positivity ranged from 74.4% in CFPA to 20.0% in SAIA, supporting previous observations that cavitary and chronic subtypes elicit stronger humoral responses compared to acute or localized forms of infection (Zhu et al., 2023; Nam et al., 2023). The notably low seropositivity in the SAIA subgroup warrants further consideration. This may be attributed to a blunted systemic immune response due to the acute and potentially more invasive nature of the infection, which can impair adequate antibody production within the disease timeframe. Furthermore, the possibility of underlying yet unidentified immunodeficiencies, not captured by a history of prolonged glucocorticoid use, could also contribute to the diminished humoral response. The low serum GM positivity (predominantly within normal ranges) contrasts with the higher sensitivity of BALF GM, particularly in AN (76%) and CCPA (60%). Notably, without a “TB-only” control group, we cannot rule out the potential impact of underlying PTB on these GM profiles, which is discussed as a study limitation. This disparity underscores the limited utility of serum GM for diagnosing non-angioinvasive forms of CPA and highlights that antigen detection is more effective when sampling the site of infection directly. These findings reinforce the diagnostic value of localized sampling and support a “clinico-radio-mycoserological” diagnostic strategy, as proposed in recent Chinese studies, especially in settings where advanced tools such as NGS remain inaccessible (van der Torre et al., 2021).

Radiologically, cavity formation (87.7%) and upper lung predominance (85.9%) emerged as hallmark features of PTB-associated CPA, consistent with global imaging standards (Hou et al., 2017; Prasad et al., 2016). CFPA demonstrated a distinct radiological profile marked by extensive fibrosis (89.7%) and emphysema (79.5%), reflecting its advanced pathological stage and aligning with previous reports identifying CFPA as the most treatment-refractory subtype (Bongomin et al., 2020). The overlap between CCPA and SAIA in clinical features (e.g., hemoptysis, fever) and imaging (e.g., cavitation) highlights the importance of microbiological confirmation to ensure accurate subtype differentiation, as previously noted in Chinese cohorts (Zhong et al., 2022).

Antifungal therapy was administered to 90% of patients, with voriconazole as the first-line agent (69.1%), consistent with international treatment guidelines (Evans et al., 2024; Sehgal et al., 2020). However, a notable 70.5% of patients received antifungal therapy for ≤6 months, which represents a critical gap in optimal care. This short treatment duration is likely multifactorial, reflecting not only challenges such as limited healthcare resources, adverse drug reactions (reported in 8.6% of cases), and insufficient follow-up, but also potential factors including suboptimal adherence and economic burden—consistent with clinical observations in similar patient populations. Supporting this, a Guangzhou-based study (Niu et al., 2020) identified low income and non-local residency as key barriers to sustained antifungal treatment, which may also contribute to the abbreviated therapy course in our cohort. Whether this short duration is further influenced by “treatment failure” endpoints (e.g., disease progression prompting therapeutic adjustment) warrants future investigation with detailed clinical outcome correlation. The CFPA subgroup exhibited the worst prognosis, with a disease progression rate of 38.5% and a mortality rate of 10.3%. In contrast, patients with AN demonstrated the highest clinical stability (92%), underscoring the prognostic significance of the CPA subtype. These findings are consistent with multicenter data identifying SAIA, older age, and low body mass index as independent predictors of mortality (Zhong et al., 2022).

Surgical intervention was performed in 19.5% of cases to manage hemoptysis or resolve diagnostic uncertainty. Combined with antifungal therapy, surgical treatment was associated with improved survival, supporting previous findings on the benefits of integrated medical-surgical management strategies (Evans et al., 2024; Kosmidis and Muldoon, 2017). However, the limited use of long-term antifungal therapy (>6 months in 3.6%) highlights a gap between guidelines and real-world practice, particularly for advanced subtypes like CFPA, where prolonged antifungal therapy is often essential to prevent disease progression and improve outcomes.

While PTB was the predominant underlying condition in this cohort, studies from low-PTB-burden regions emphasize chronic non-mycobacterial pulmonary diseases (e.g., COPD) as primary risk factors (Maitre et al., 2021), reflecting distinct etiological patterns. The high *Aspergillus* IgG positivity in cavitary subtypes aligns with data from Taiwan identifying IgG as a key diagnostic marker (Lai and Hsueh, 2024). However, regional validation of antibody cutoff thresholds remains necessary to improve diagnostic specificity.

The single-center, retrospective design of this study limits the generalizability of the findings, particularly to CPA cases unrelated to PTB. Future research should focus on establishing standardized treatment endpoints to reduce variability in assessing therapeutic success or failure, conducting prospective multi-center studies to validate subtype-specific prognostic factors and refine antifungal regimens, and developing resource-adapted diagnostic tools such as point-of-care serological assays to enhance early and accurate CPA detection in PTB-endemic regions.

## Conclusion

PTB-associated CPA in China presents with distinct subtype-specific clinical and radiological patterns, with CCPA as the most prevalent form and CFPA as the most severe. Accurate diagnosis requires the integration of clinical symptoms, imaging findings, and microbiological evidence. Treatment outcomes are shaped by disease subtype, underlying comorbidities, and healthcare resource availability. The findings highlight the need for region-specific, evidence-based guidelines and multidisciplinary management strategies to improve outcomes in high-PTB-burden areas, where CPA remains a significant yet underrecognized public health concern.

## Data Availability

The raw data supporting the conclusions of this article will be made available by the authors, without undue reservation.
