# Supplementary material for: Clinical features, diagnostic test performance, treatment and outcome of pulmonary tuberculosis patients with chronic pulmonary aspergillosis in China: a retrospective, observational study
Source: Front Cell Infect Microbiol. 2026 Jan 20;15:1653842. doi: 10.3389/fcimb.2025.1653842 (PMC12864492; doi:10.3389/fcimb.2025.1653842)
Supplement: Supplementary file 1 [file DataSheet1.docx]

**Supplement Figures**

**
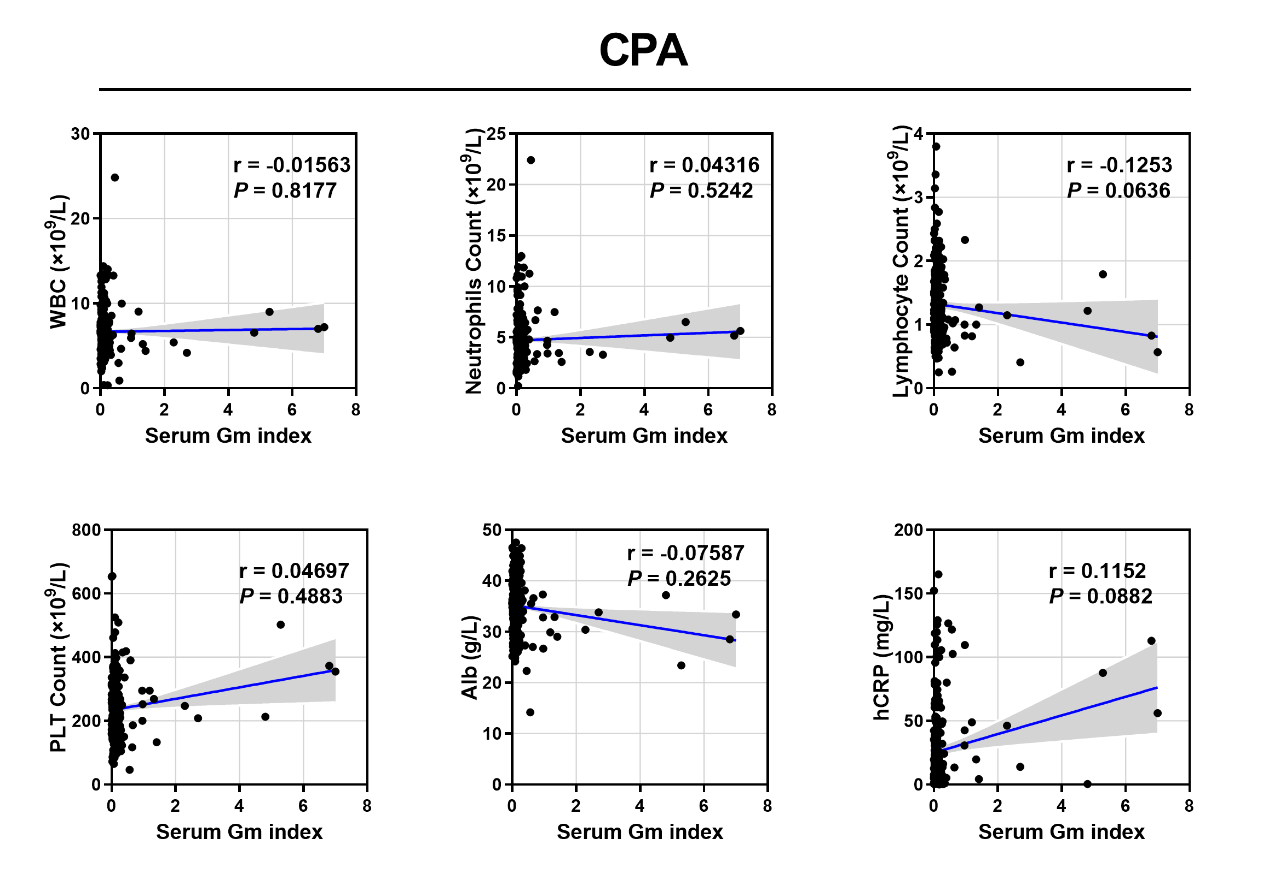
**

**Supplementary Figure S1. Correlation analysis between serum GM index and peripheral blood laboratory parameters in pulmonary tuberculosis (PTB) patients with chronic pulmonary aspergillosis (CPA).** GM, galactomannan; WBC, white blood cell; PLT, blood platelet; ALB, albumin; CRP, C-reactive protein.

**
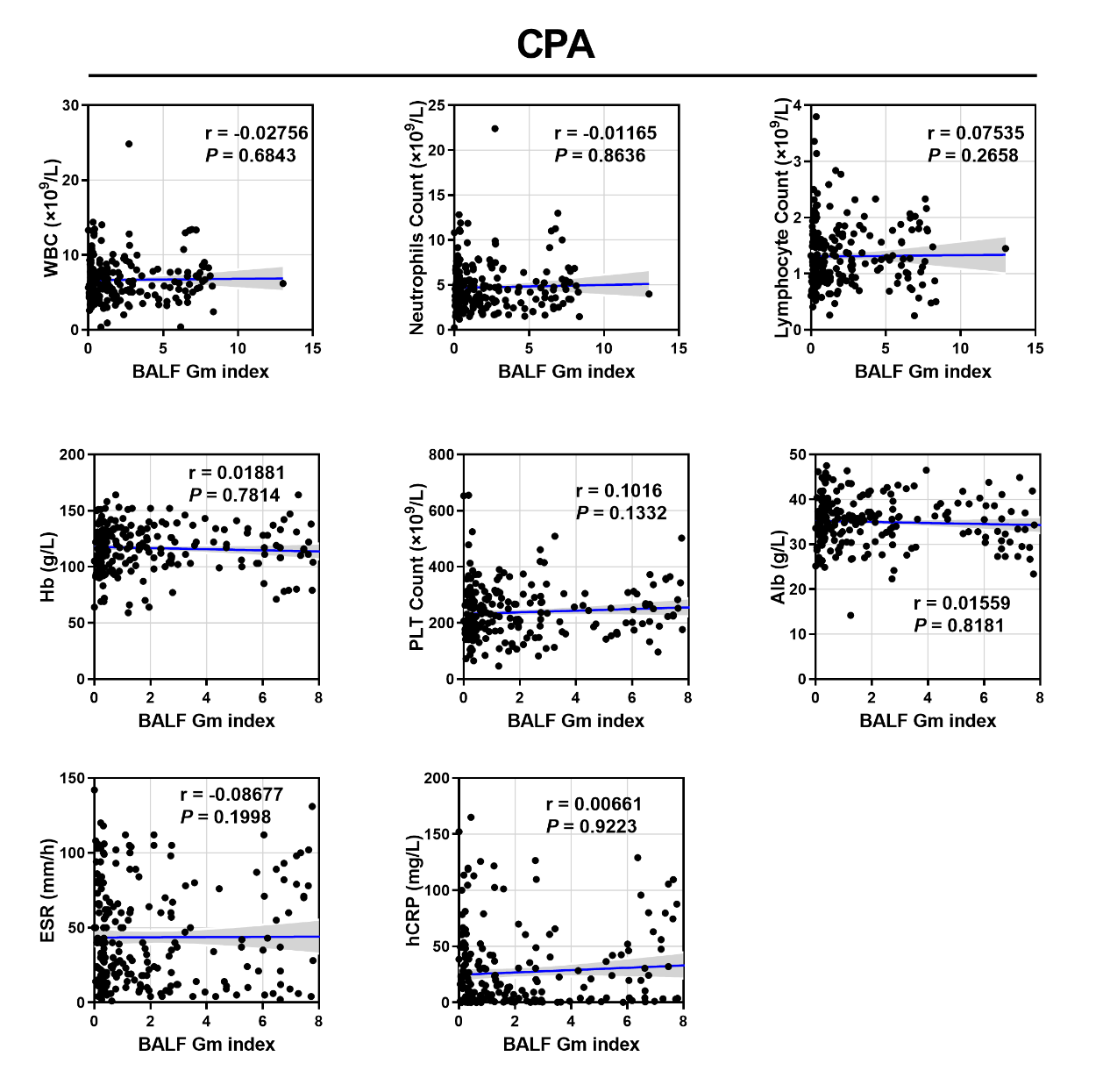
**

**Supplementary Figure S2. Correlation analysis between BALF GM index and peripheral blood laboratory parameters in PTB patients with CPA.** BALF, bronchoalveolar lavage fluid; Hb, hemoglobin; ESR, erythrocyte sedimentation rate.


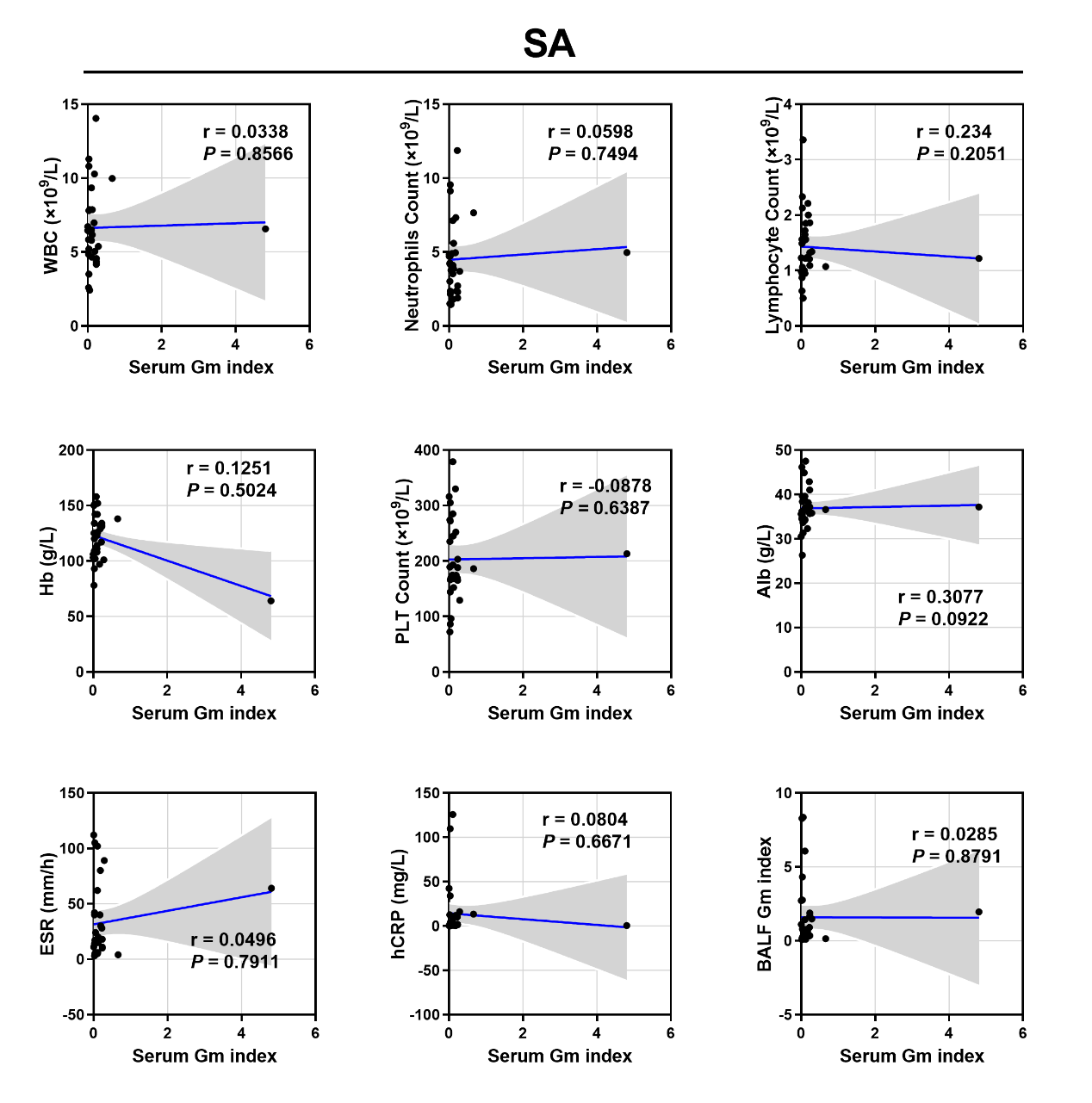


**Supplementary Figure S3. Correlation analysis between serum GM index and peripheral blood laboratory parameters in PTB patients with simple aspergilloma (SA).**


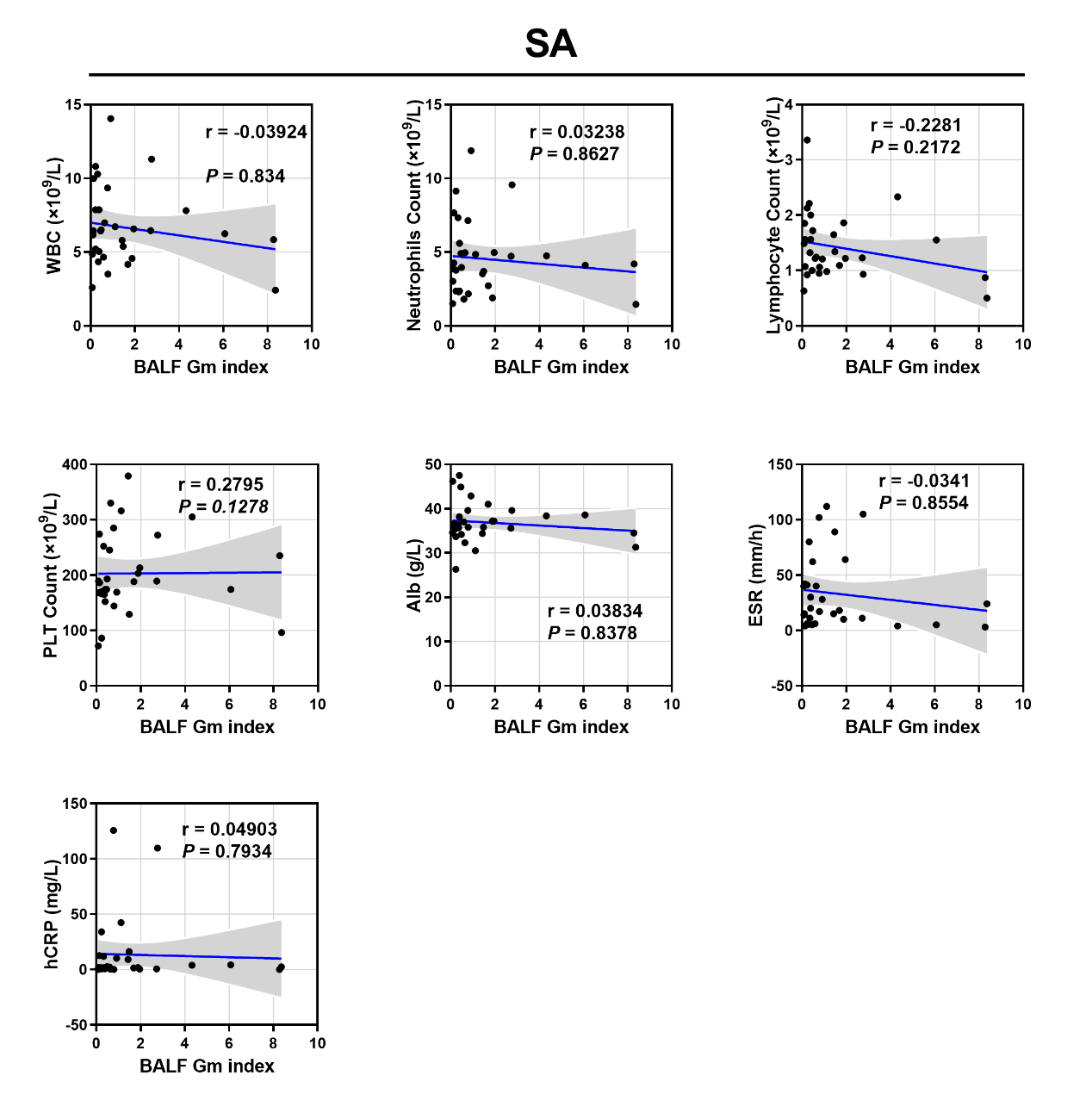


**Supplementary Figure S4. Correlation analysis between BALF GM index and peripheral blood laboratory parameters in PTB patients with SA.**

**
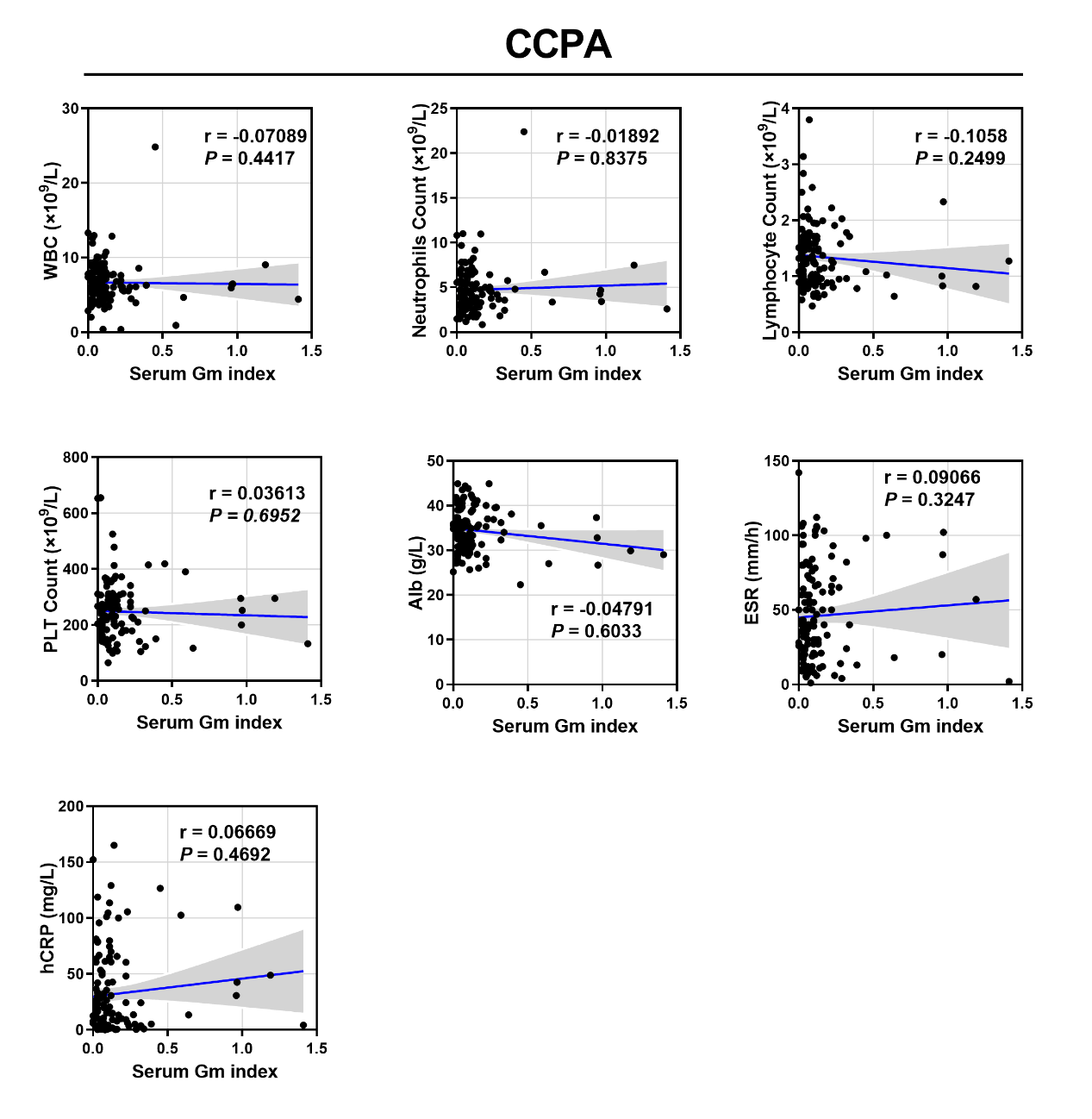
**

**Supplementary Figure S5. Correlation analysis between serum GM index and peripheral blood laboratory parameters in PTB patients with chronic cavitary pulmonary aspergillosis (CCPA).**


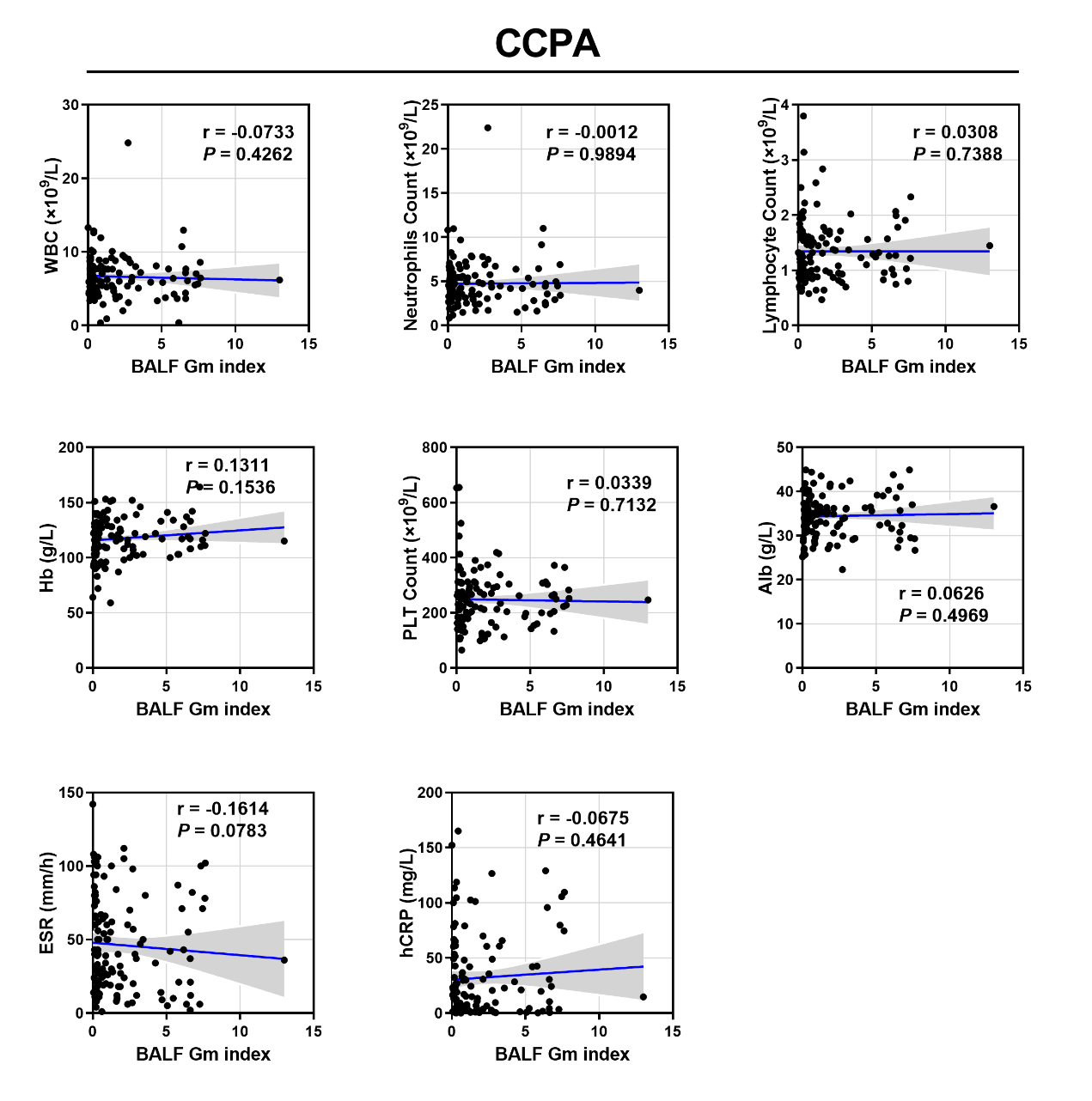


**Supplementary Figure S6. Correlation analysis between BALF GM index and peripheral blood laboratory parameters in PTB patients with CCPA.**

**
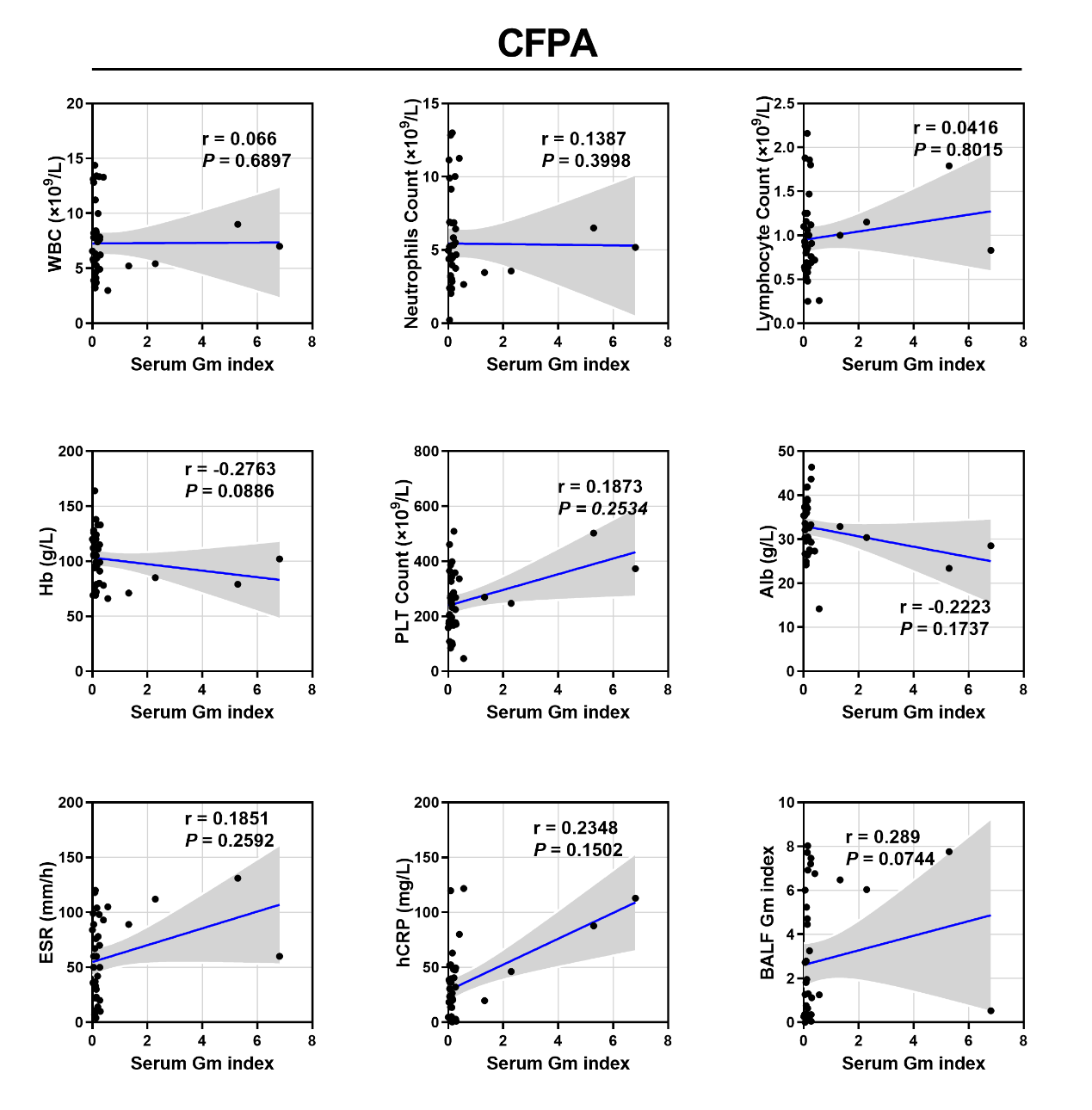
**

**Supplementary Figure S7. Correlation analysis between serum GM index and peripheral blood laboratory parameters in PTB patients with chronic fibrosing pulmonary aspergillosis (CFPA).**


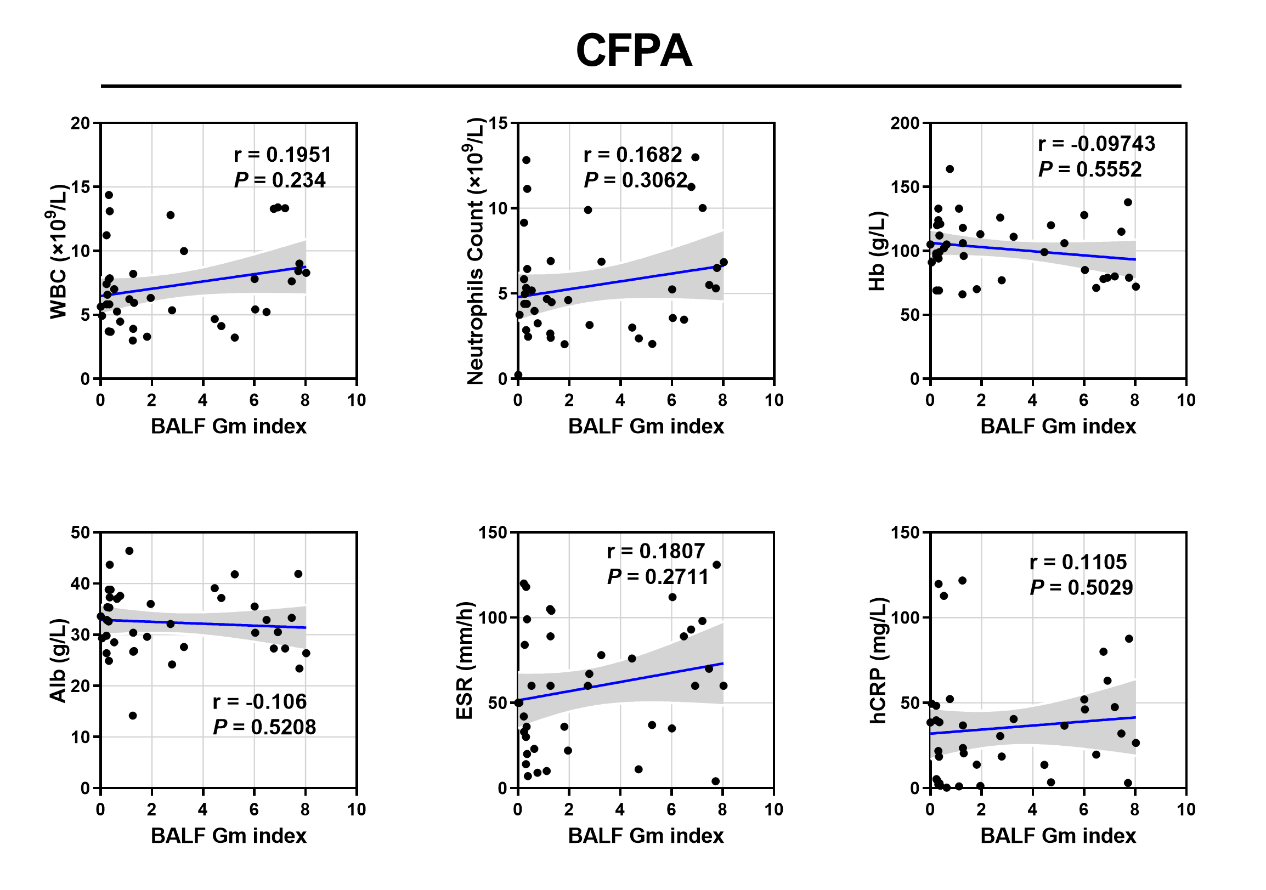


**Supplementary Figure S8. Correlation analysis between BALF GM index and peripheral blood laboratory parameters in PTB patients with CFPA.**

**
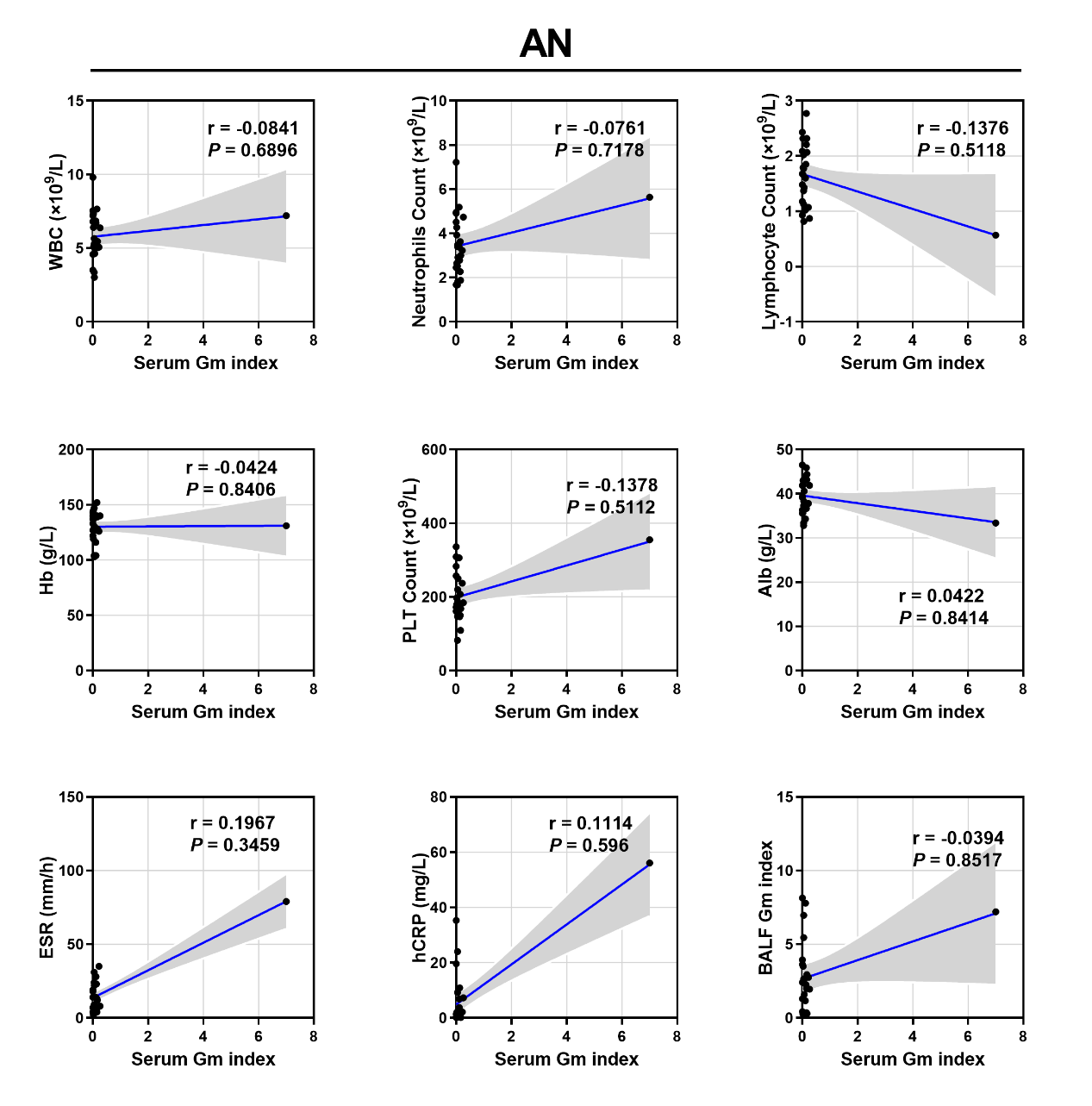
**

**Supplementary Figure S9. Correlation analysis between serum GM index and peripheral blood laboratory parameters in PTB patients with *Aspergillus* nodule (AN).**

**
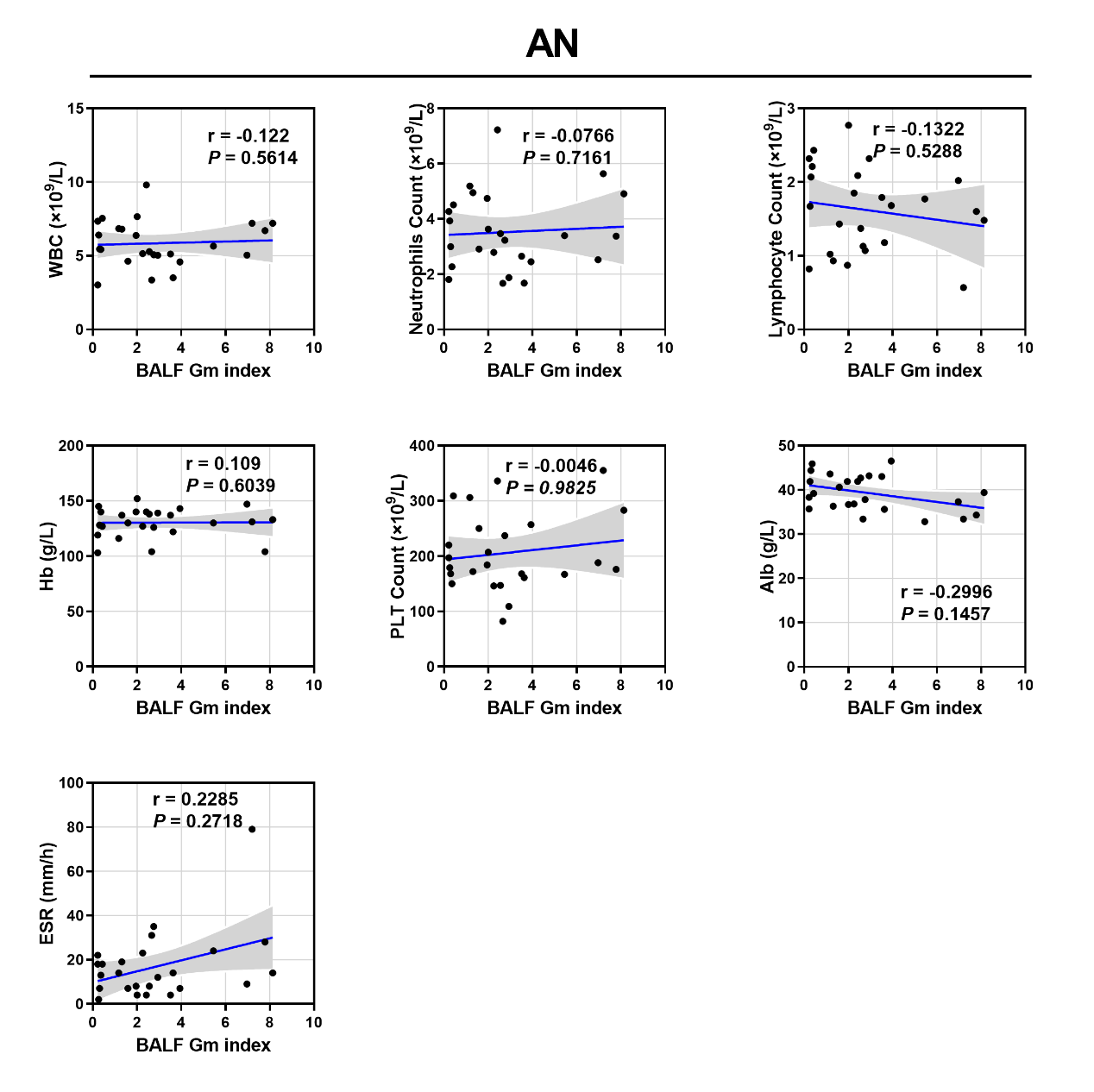
**

**Supplementary Figure S10. Correlation analysis between BALF GM index and peripheral blood laboratory parameters in PTB patients with AN.**

**
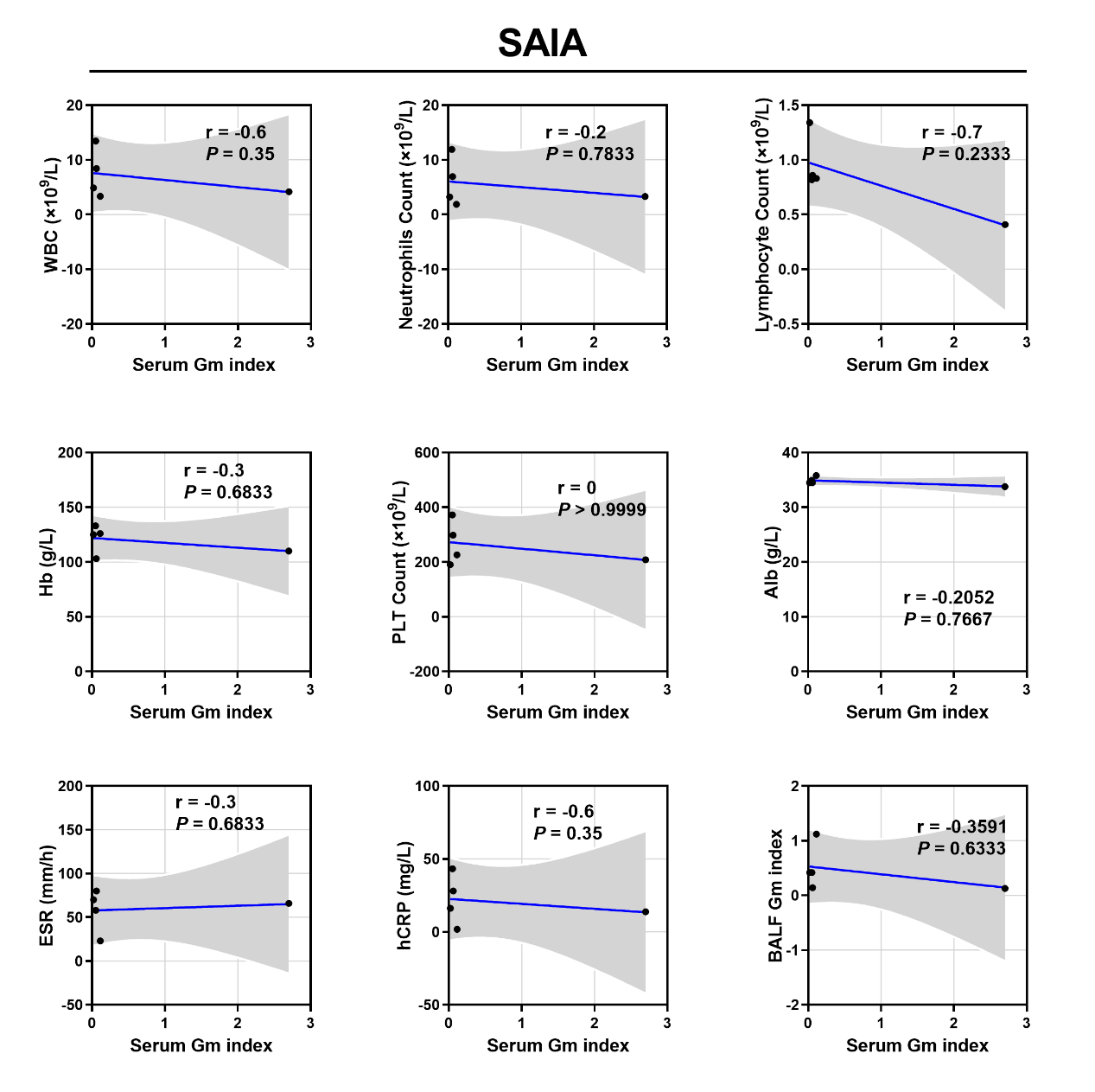
**

**Supplementary Figure S11. Correlation analysis between serum GM index and peripheral blood laboratory parameters in PTB patients with semi-invasive aspergillosis (SAIA).**

**
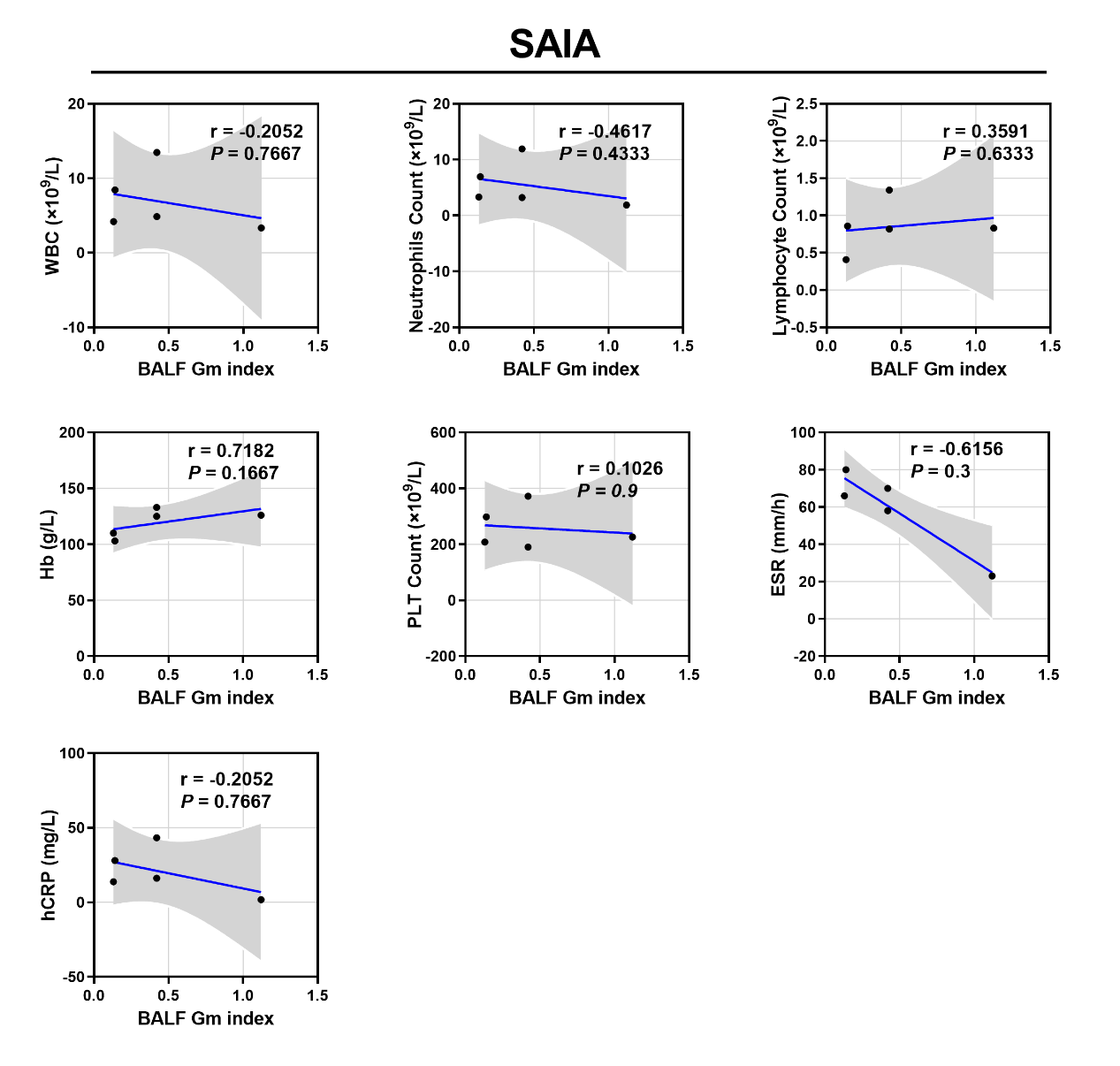
**

**Supplementary Figure S12. Correlation analysis between BALF GM index and peripheral blood laboratory parameters in PTB patients with SAIA.**
